# Potential of *Syzygnium polyanthum* as Natural Food Preservative: A Review

**DOI:** 10.3390/foods12122275

**Published:** 2023-06-06

**Authors:** Nur Julizan, Safri Ishmayana, Achmad Zainuddin, Pham Van Hung, Dikdik Kurnia

**Affiliations:** 1 Department of Chemistry, Faculty of Mathematics and Natural Science, Universitas Padjadjaran, Sumedang 45363, Indonesia; 2 School of Biotechnology, International University, Vietnam National University, Ho Chi Minh City 721400, Vietnam

**Keywords:** *Syzygnium polyanthum*, antioxidant, antimicrobials, natural preservative, food preservation

## Abstract

Food preservation is one of the strategies taken to maintain the level of public health. Oxidation activity and microbial contamination are the primary causes of food spoilage. For health reasons, people prefer natural preservatives over synthetic ones. *Syzygnium polyanthum* is widely spread throughout Asia and is utilized as a spice by the community. *S. polyanthum* has been found to be rich in phenols, hydroquinones, tannins, and flavonoids, which are potential antioxidants and antimicrobial agents. Consequently, *S. polyanthum* presents a tremendous opportunity as a natural preservative. This paper reviews recent articles about *S. polyanthum* dating back to the year 2000. This review summarizes the findings of natural compounds presented in *S. polyanthum* and their functional properties as antioxidants, antimicrobial agents, and natural preservatives in various types of food.

## 1. Introduction

Food preservation is necessary to define and enhance food shelf life, customer acceptance, and increase food security [1]. The preservation of food by means of the addition of chemicals is one way. Synthetic chemical preservatives are used widely in the food industry to inhibit the deterioration caused by microbial growth, enzyme activity, and oxidation. Due to the potential health risks posed by synthetic preservatives in food, however, consumers are becoming hesitant to use such products [2]. Oxidation and pathogen microbial growth are the primary causes of food deterioration [3,4]. Therefore, food preservation is performed to prevent food spoilage.

In Indonesia, various medicinal plants have been used as traditional treatments for a variety of degenerative disorders for generations. *Syzygnium polyanthums* are easily accessible evergreen trees. *S. polyanthum*, also known as “Salam”, has comestible fruits and flowers, and its dried leaves are used in numerous Indonesian dishes as a food additive [5] or as essential seasoning in Indonesian cuisine [6]. It is prevalent in Cambodia, Indonesia, Malaysia, Myanmar, Thailand, and Vietnam [7]. The polyphenols, flavonoids, and tannins found in *S. polyanthum* serve as antioxidants and antimicrobial agents [8,9,10]. For preventing food deterioration, *S. polyanthum* is an alternative natural preservative to synthetic preservatives, which are unfavorably considered by consumers [11,12,13]. This article summarizes the chemical composition and impact of *S. polyanthum* on the oxidation and microbial activity of various foods and preservation techniques.

## 2. Food Spoilage

Lipid oxidation and microbial activity are major factors in food degradation [14]. Periodically, the quality of the food continues to deteriorate. Mainly, food quality degradation is caused by chemical, biochemical, or physical changes [15]. Various definitions of food spoilage exist. In general, food spoilage indicates conditions that are inappropriate for consumption. Regarding food safety, where food spoilage may result in infection or even mortality, the issue of food spoilage becomes crucial [16]. Food spoilage can occur along the processes animal farming, transportation, butchering, processing, wrapping, delivery, and home meal preparation [17,18].

Rigor mortis, which lasts approximately 12 h at room temperature in tropical climates, can immediately cause the spoilage of fresh meat. Rigor mortis is the process of losing flexibility as a result of tightening after death. Typically, digestive enzymes and lipases, microbiological contamination, and oxidation degrade meats. During the process of meat contamination, various components decompose, and new compounds are formed. This new compound changes the aroma, flavor, and texture of the meat [19,20,21]. The food industry continues to pursue alternative technologies for quality assurance, livestock safety, and long-term preservation in order to meet the demands of modern consumers for wholesome meat-based products [22]. Oxidation and microbial contamination not only cause food spoiling in fresh meat products but also in oil, paste, fruit, fruit products, vegetables, veggies products, and bakery products [23,24].

Lipid oxidation of foods results in the formation of harmful compounds, has a negative impact on the sensory qualities of foods, and is a major contributor to reducing shelf life and making food waste [25]. As a result of metabolic activity, the growth of microorganisms in food creates variations in the physical appearance of food. Some of these changes result in food spoilage and food poisoning. The most critical elements that affect microbial development in foods are the intrinsic and extrinsic environment of food storage [26,27]. Food spoilage due to oxidation and microbial activity is explained further in the next section. 

### 2.1. Oxidation Activity

Food materials derived from plants and animals generally consist of water, fat, protein, and carbohydrates as the main building components [28]. The most prevalent lipid molecules in nature are fats and oils. At normal temperature, fat is solid while oil is liquid, suggesting a distinction in their consistency and physical qualities. Differences in the number of carbon chain lengths, double bonds, and cis or trans forms of unsaturated fatty acids determine the melting point of fats (polyunsaturated fatty acids, PUFA) [29]. Prior research indicates that the consumption of PUFAs has significant health benefits. These include the α-linolenic acid (ALA 18:3 ω-3), eicosapentaenoic acid (EPA, 20:5 ω-3), docosapentaenoic acid (22:5 ω-3), docosahexaenoic acid (DHA, 22:6 ω-3), γ-linolenic acid (GLA, 18:3 ω-6), and arachidonic acid (ARA, 20:6 ω-6) [30]. Long-chain ω-3 PUFA provide significant health benefits, particularly for the prevention of cardiovascular and certain inflammatory diseases [31]. Food products with high levels of DHA and EPA are essential for pregnant women [32]. In foods, a lipid can serve as an enzyme, flavoring, and coloring agent [33]. 

Oxidation is the main cause of food degradation. Lipids contain two main groups, namely polar lipids (phospholipids) and neutral lipids (triglycerides), and they are easily oxidized by reacting with reactive oxygen precursors and free radicals. This oxidation can cause rancidity, discoloration, decreased nutritional quality, and some toxic compounds [34]. PUFAs are the main target of attack by reactive oxygen species (ROS), leading to a nonenzymatic oxidation process, called lipid peroxidation [35]. Lipid oxidation associated with a decrease in the quality of fatty foods occurs based on the mechanisms of auto-oxidation, photo-oxidation, and enzymatic oxidation. Photo-oxidation produces aliphatic and aromatic oxidation compounds, due to the presence of a light photosensitizer, which converts triplet oxygen into singlet oxygen, which is an extremely reactive and nonradical molecule. The PUFA oxidation assisted by lipoxygenase enzymes through free radical mechanisms forms specific hydroperoxides [34,36]. Hydroperoxides formed through enzymatic oxidation processes are intermediates in synthesis of prostaglandins and eicosanoids [37]. Among these mechanisms, auto-oxidation, which is a persistent chain reaction involving free radicals, is the most typical and challenging reaction to avoid [38]. 

**Figure 1:**
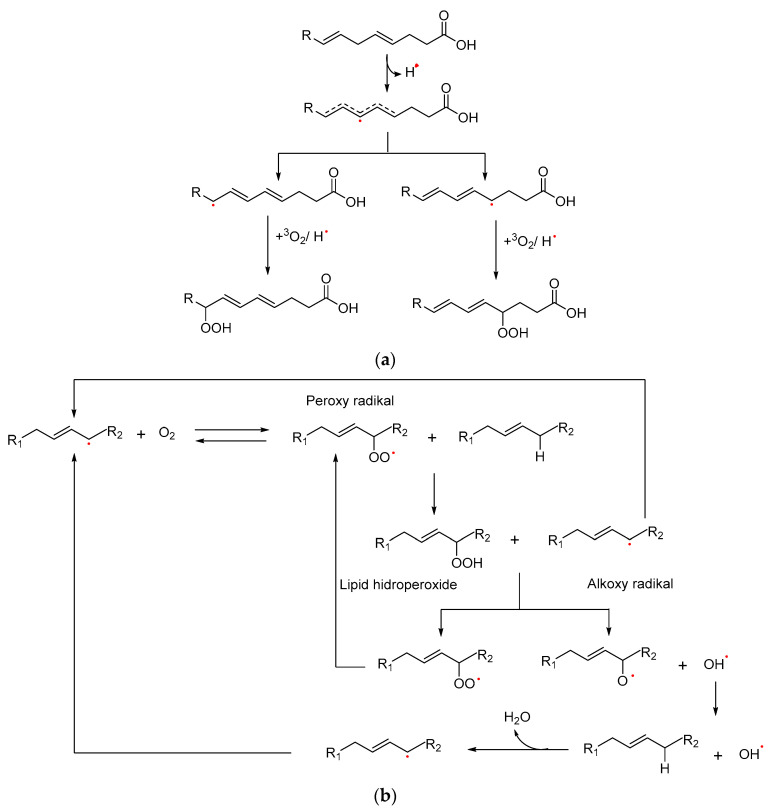

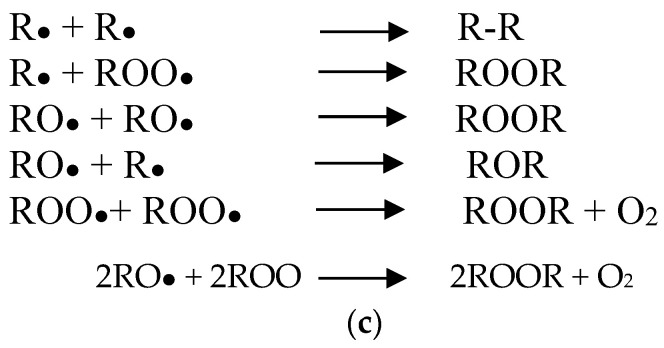
Lipid auto-oxidation stages [36,42,45]. (**a**) Initiation; (**b**) Propagation; (**c**) Termination.

Auto-oxidation occurs through three stages: initiation, propagation, and termination [39,40]. During the initiation stage, a hydrogen atom is abstracted from its adjacent carbon into a double bond in the unsaturated fatty acid (RH), forming an alkyl radical (R•). The alkyl radical product often undergoes double-bound rearrangement to stabilize itself into a conjugated diene or triene [41,42]. During the propagation step, peroxyl radicals, which are ordinarily generated as primary products, abstract hydrogen from a nearby lipid (atom transfer process). The new alkyl radical combines with molecular oxygen once more to produce new peroxyl radicals, and the cycle is continued. Before two R• unite to complete the process, these propagation processes may repeat up to 100 times [39,43,44]. The termination stage involves a reaction between two radical molecules to produce a nonradical molecule [36]. The lipid auto-oxidation stages are illustrated in Figure 1.

The mechanism for the generation of secondary oxidation products such as these volatile chemicals is extremely complicated and varied depending on the PUFA substrate [46]. Auto-oxidation involves the reaction of triplet oxygen with organic compounds under moderate conditions. Fat auto-oxidation is a series of reactions involving free radicals that occur in three stages, namely initiation, propagation, and termination [47]. A number of secondary oxidation products including volatiles can be formed from the early stages of EPA and DHA oxidation; meanwhile, only a small number of products were detected in the first stage of linolenic acid (LA) oxidation. This observation occurs because EPA- and DHA-LOOH are less stable than LA-LOOH. Even at very low oxidation levels, fish oil that is rich in EPA and DHA frequently has a fishy and metallic flavor [48]. Fat oxidation can be monitored through the formation of primary and secondary reaction products. The primary products of fat oxidation can be observed to form hydroperoxides and conjugated dienes. Secondary products that can be measured are anisidine values, TOTOX values, and volatile compounds [49].

### 2.2. Microbial Activity 

Food spoilage is a change in food quality that renders it unpleasant and unsafe for human or animal intake. The presence of odors and changes in texture are indicators of food spoilage. Microorganisms are the primary cause of food spoilage [50]. Microbial pathogens in food can cause spoilage and contribute to the incidence of foodborne disease, and the emergence of multidrug-resistant and disinfectant-resistant bacteria (such as Staphylococcus aureus, Escherichia coli, and Pseudomonas aeruginosa) has increased rapidly, resulting in an increase in morbidity and mortality [51].

Through the production of toxic gases and volatile organic compounds, bacterial metabolism, and the accumulation of extracellular chemicals, bacteria contaminate food [52]. In the meat and poultry industries, bacterial spoilage causes color changes, a miserable odor, and slimy foods [53]. Psychotropic Pseudomona is one of the primary organisms responsible for the spoilage of fresh protein foods such as meat and fish that have been stored aerobically. This is due in part to its capacity to produce extracellular proteolytic and lipolytic enzymes and to produce slime on food surfaces [54]. *Leoconostoc, Pediococcus damnosus*, *Pseudomonas*, *S. putrefaciens*, *S. phosporeum Aeromonas* spp., *Enterobacteriaceae*, *B. cereus*, *Salmonella species*, *Listeria monocytogenes*, *Staphylococcus aureus*, *Campylobacter species*, *Bacillus cereus*, and *Escherichia coli* are the most common bacterial causes of foodborne diseases [55,56]. However, by sacrificing the metabolic efficiency of aerobic or anaerobic electron transfer chains, lactic acid bacteria can adhere to a “iron-free diet” and occupy ecological niches associated with plants and animals in which iron deficiency inhibits growth. Lactic acid bacteria dominate the fermentation microbiota of the majority of fermented foods, but they also play an important role as spoilage organisms [57].

The microbial degradation of aquatic food products by spoilage microorganism is linked to the release of chemicals/gases that impart an off-flavor and odor, indicating spoilage. As biochemical indicators of fish quality, these volatile amines, primarily di-methylamine, trimethylamine, hydrogen sulfide, and ammonia, have an unpleasant taste. Fish typically contains a high concentration of free amino acids. Microorganism convert amino acids and urea into ammonia, biogenic amines, organic acids, and sulfur compounds, while trimethylamine oxide is decomposed into trimethylamine and dimethylamine, which significantly degrades the quality of fish [24]. 

Fungi are a group of microorganisms that cause severe spoilage and are resistant to most food industry preservation procedures. Filamentous molds and yeast are very easy to contaminate and disperse in food at the processing stage. As a result of their varied structure, in general, fungi are able to survive in certain ecological systems so that they easily contaminate commercially processed foods. Several indicators of food spoilage are used to determine the shelf-stability of foods [58,59]. Since a single microbial criterion may not accurately predict the shelf life and spoilage condition of meats, consider the following parameters for identifying microbiological spoilage indicators: (1) indicators must be present in low numbers in the fresh product, (2) the amount of these microorganisms must increase dramatically from farm to fork, (3) such organisms must be the predominant cause of deterioration at the outset of spoilage, and (4) the spoilage features owing to these pathogens must be simply and immediately detected [60]. Food spoilage caused by fungi infection can be seen by the formation of black, white, or pink mold [61,62,63], congested filamentous appearance [64], gas production that can occasionally cause an explosion, and defection of food surface [58].

Food poisoning is an effect of bacteria-contaminated food consumption [65]. In general, bacterial food poisoning causes gastrointestinal disease [66]. In the EU, nearly half of all cases of diarrhea due to contamination by Shiga toxin-producing Escherichia coli require hospitalization [67]. Food poisoning in *Salmonella* causes salmonellosis [68]. S. aureus staphylococcal enterotoxins frequently cause staphylococcal food poisoning during food contamination. The severity of the illness is dependent on the quantity of toxins consumed and the consumer’s overall health. Some report nausea and vomiting, while others report diarrhea, prostration, and fever [69].

## 3. Food Preservation

Food preservation is described as the methods used to minimize internal and external variables that could cause food to decay. The basic purpose of food preservation is to extend the shelf life of food while preserving its original nutritional texture, color, and value. Food preservation techniques can be classified into three groups, namely physical processing, biological processing, and chemical processing [70]. Physical preservation processes can be thermal or nonthermal techniques. Thermal preservation techniques such as pasteurization, high-temperature sterilization, drying, and evaporation are widely used in conventional food preservation. These techniques can inhibit the microbial activity that leads to food spoilage, but they can also modify the physical, sensory, and nutritional properties of foods, such as heat-sensitive vitamins and polyphenols [71,72]. The food is subjected to nonthermal treatment for a very brief amount of time and is treated at room temperature. Since the exposure period is brief and the temperature is low, there is no risk of heat-sensitive nutritional components in the food being lost, no risk of texture damage, and no risk of the development of any toxin in the food. Several methods that can be used as nonthermal preservation techniques, such as ultrasonication, cold plasma technology, supercritical technology, irradiation, pulsed electric field, high hydrostatic pressure, pulsed ultraviolet technology, and ozonation [73,74]. Impressively, nonthermal technologies are confirmed to not only preserve bioactive compounds better than thermal treatments, but in some cases, they can also induce their release from the living cells contained in the product, resulting in an increase in their interactively detectable concentration and, frequently, their bioaccessibility [75]. 

Another method of preservation is biological processing. The biopreservation process is a method of food preservation that utilizes the antibacterial power of naturally existing organisms and their metabolites [76]. One of the methods of biopreservation is fermentation. Fermentation reduces food spoilage and rids food of pathogenic germs and metabolites through the production of numerous beneficial byproducts with bacteria [77]. Fermentation will generate high-quality products with certain improved nutritional values and an abundance of bioactive compounds. Additionally, fermentation results in the inclusion of several probiotics. Therefore, fermentation will generate raw materials into new products with improved nutritional value, intestinal health, and certain biological functions [78].

Using chemical reagents to preserve food is one of the oldest and most conventional practices. The effectiveness of this method depends on the amount and specificity of the chemical reagents, as well as the physical and chemical properties of the food. Chemical preservation methods involve the inclusion of chemical preservatives and pH regulation [70,79]. In the food and pharmaceutical industries, synthetic preservatives such as sodium acetate, sodium benzoate, potassium sorbate, and butyl paraben are commonly employed [80]. Parabens are alkyl esters of *p*-hydroxybenzoic acid that possess antibacterial, antifungal, and preservation characteristics [81]. Sodium benzoate is commonly used to preserve margarines, sauces, marmalades, gelatin, liqueurs, beers, and fruit juices. Utilization of sodium benzoate in excess of the threshold can result in genotoxic, clastogenic, and neurotoxic effects [82]. Synthetic preservatives have numerous detrimental consequences on human health. In recent years, people have demanded the complete replacement of chemically manufactured preservatives due to their detrimental effects on health. This has led to a growing interest in the development of more natural alternatives to extend the shelf life and safety of food [83,84]. As traditional preservatives, natural chemicals such as salt, sugar, vinegar, alcohol, and diatomaceous earth are also employed. Certain plants have their own preservatives, such as citric and ascorbic acids from lemon or other citrus juice, which prevent the activity of the phenolase enzyme responsible for the browning of cut apple and potato surfaces [85]. According to numerous research studies, plant oils and plant extracts have been utilized as food preservatives and medical therapeutic agents for decades [83]. Mulberry and onions are used to preserve fish to maintain its sensory attributes [86]. Oil of bay, clove, cinnamon and thyme are used widely as food preservatives [87,88]. Cinnamon is a heat-stable food preservative that can be used in cookies [89]. Some plants such as mint leaves, oregano, grape seed, clove, mustard, and black currant can be used as meat preservatives [90].

## 4. Syzygnium Polyanthum

*S. polyanthum* leaves are widely used as traditional medicine and food seasoning, especially in Indonesia and Malaysia [91,92]. *S. polyanthum* is classified as *Plantae* kingdom, *Magnollophyta* division, *Magnoliopsida* class, *Myrtalles* ordo, *Myrtaceae* family, and *Syzygium* genus [93]. *S. polyanthum* has a taproot and a round, silky trunk. It has an oval shape, a sharp base and a pointed tip, flat edges, and a dark green upper surface and a lighter green lower surface. Its leaves emit a pleasant aroma when crushed [94]. *S. polyanthum* is widely used as a traditional remedy for a range of infections, such as diabetes [95], cardiovascular disease [96], hyperlipidemia [97], anemia [98], kidney disease [99], liver disease [100], gout [101], oxidative disease [102], and some bacterial diseases [103].

Numerous secondary metabolites are abundant in *S. polyanthum* leaves. Major secondary metabolites are 28% tannin group and its derivates, followed by 25% phenolic groups [10]. *S. polyanthum* leaves have various bioactivities, including antioxidant, antibacterial, anticancer, and antidiabetic substances [104]. 

**Figure 2:**
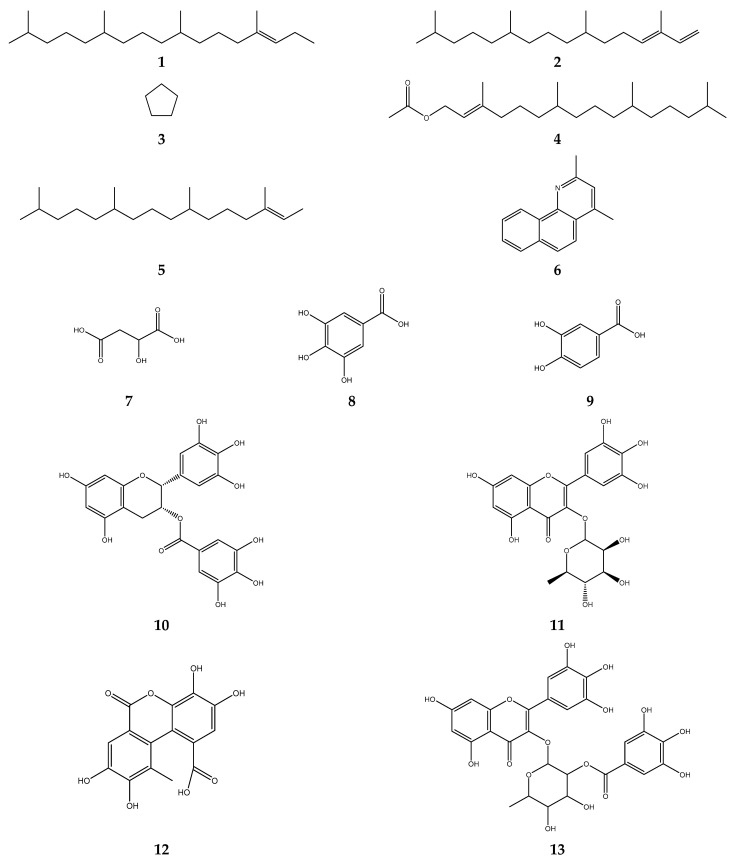
The compounds in S. polyanthum ethanol extract: (**1**) phytol; (**2**) (Z)-1,3-phytadiene; (**3**) cyclopentane; (**4**) phytol acetate; (**5**) 2-hecadecene,3,7,15-tetramethyl; (**6**) cis-1,3-dimethyl-4-aza phenanthrene; (**7**) malic acid; (**8**) gallic acid; (**9**) protocatechuic acid; (**10**) epigallocatechin gallate; (**11**) myricetin-3-O-rhamnoside; (**12**) luteic acid; and (**13**) desmanthin-1.

Compounds found in *S. polyanthum* ethanol extract are phytol, (Z)-1,3-phytadiene, cyclopentane, phytol acetate, 2-hecadecene,3,7,15-tetramethyl, and *cis*-1,3-dimethyl-4-aza phenanthrene [105]. Meanwhile, compounds found in fraction acetone:water (4:1) are malic acid, gallic acid, protocatechuic acid, epigallocatechin gallate, myricetin-3-O-rhamnoside, luteic acid, and desmanthin-1 [106]. The structure of *S. polyanthum* extract compounds is shown in Figure 2. 

**Figure 3:**
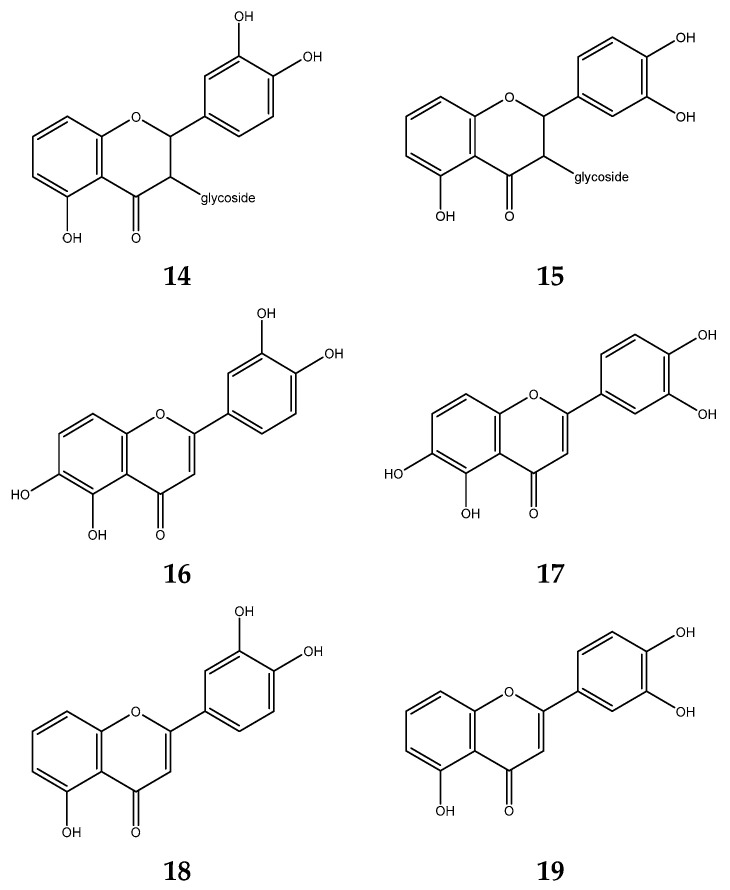
Flavonoid compounds of S. polyanthum: (**14**) 5,3′,4′-trihydroxyflavone-3-C-glycoside; (**15**) 5,4′,5′-trihydroxyflavone-3-C-; (**16**) 5,6,3′,4′-tetrahydroxyflavone; (**17**) 5,6,4′,5′-tetrahydroxyflavone; (**18**) 5,3′,4′-trihydroxyflavone; and (**19**) 5,4′,5′-trihydroxyflavone.

The primary secondary metabolites in *S. polyanthum* water extract are phenol hydroquinone, tannins, and flavonoid. Flavonoid extracts of *S. polyanthum* exhibited the highest antioxidant activity of the three selective metabolites [107]. Flavonoid compounds detected on *S. polyanthum* are 5,3′,4′-trihydroxyflavone-3-C-glycoside; 5,4′,5′-trihydroxyflavone-3-C-glycoside; 5,6,3′,4′-tetrahydroxyflavone; 5,6,4′,5′-tetrahydroxyflavone and 5,3′,4′-trihydroxyflavone or 5,4′,5′-trihydroxyflavone [108]. The structure of *S. polyanthum* flavonoid compounds is shown in Figure 3.

**Figure 4:**
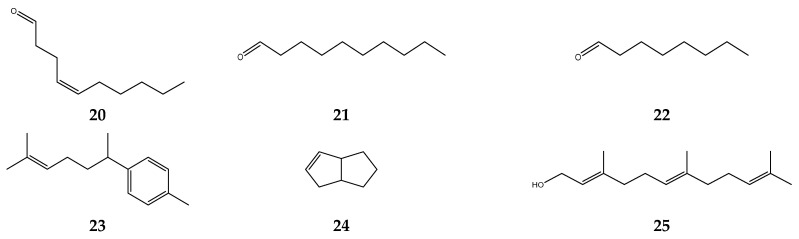

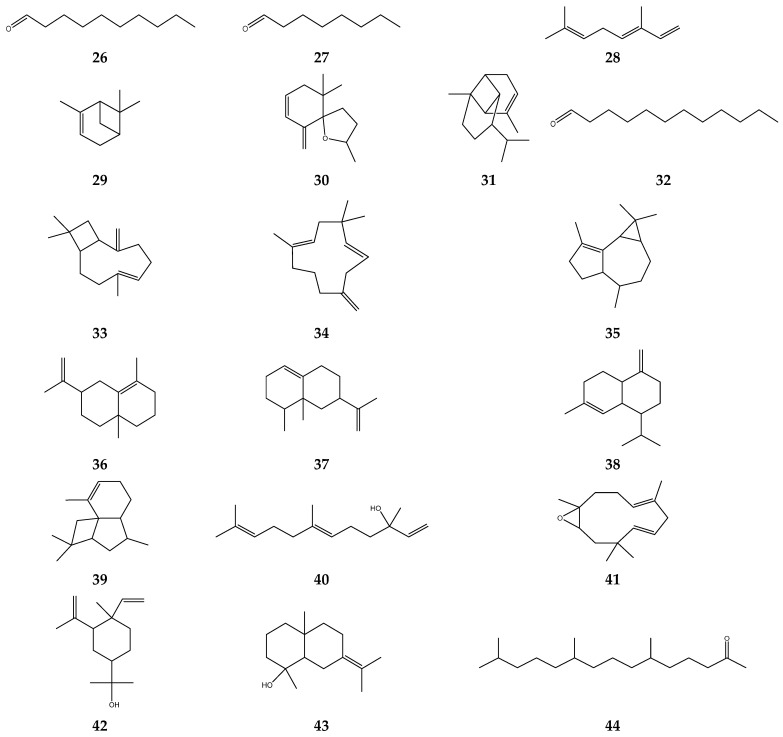
The compounds in S. polyanthum essential oil: (**20**) cis-4-decenal; (**21**) 1-decyl aldehyde; (**22**) capryl aldehyde; (**23**) α-curcumene; (**24**) 1,2,3,3a,4,6a-hexahydropentalene; (**25**) farnesol; (**26**) decanal; (**27**) octanal; (**28**) β-ocimene; (**29**) α-pinene; (**30**) vitispirane; (**31**) α-copaene; (**32**)dodecanal; (**33**) trans-caryophyllene; (**34**) α-humulene; (**35**) α-gurjunene; (**36**) 4,11-selinadiene; (**37**) valencene; (**38**) γ-cadinene; (39) α-panasinsene; (**40**) nerolidol; (**41**) humulene oxide; (**42**) elemol; (**43**) juniper camphor; and (**44**) hexahydrofarnesyl acetone.

*S. polyanthum* can be utilized not only as an extract but also as an essential oil. Polar and nonpolar compounds are essential oil building mixtures consisting of 20–60 compounds in various concentration. Nevertheless, there are just two or three major compounds and trace amounts for the others. The compounds of *S. polyanthum* are *cis*-4-decenal, 1-decyl aldehyde, capryl aldehyde, α-curcumene, 1,2,3,3a,4,6a-hexahydropentalene, octanal, decanal, farnesol, β-ocimene, α-pinene, vitispirane, α-copaene, dodecanal, *trans*-caryophyllene, α-humulene, α-gurjunene, 4,11-selinadiene, valencene, γ-cadinene, α-panasinsene, nerolidol, *n*-humulene oxide, elemol, juniper camphor, and hexa-hydrofarnesyl acetone [109,110,111,112]. The structure of *S. polyanthum* essential compounds is shown in Figure 4.

## 5. Bioactivities of *S. polyanthum* Leaves 

### 5.1. Antioxidant Activity of S. polyanthum Leaves

Some research laboratories have developed synthetic antioxidants that replace phenols and aromatic amines, such as butylated hydroxyanisole (BHA), butylated hydroxytoluene (BHT), and propyl gallate (PG) [113]. The community prefers natural ingredients and is extremely concerned about the negative perceptions of the safety of synthetic food ingredients. Consequently, numerous sectors are interested in effective natural antioxidants [114,115]. 

Antioxidants typically prevent the initiation of lipid auto-oxidation. During food processing and storage, antioxidants such as tocopherol and astaxanthin can minimize the adverse effects of oxidative stress, because they act as scavengers of ROS and reactive oxygen metabolites (ROMs) species [116]. Antioxidants are categorized as either primary or secondary. Primary antioxidants are antioxidants that can inhibit or delay oxidation in two ways: by scavenging free radicals or by inhibiting lipid peroxidation [117]. Secondary antioxidants slake singlet oxygen, dissolved peroxides, chelate prooxidative metal ions, and suppress oxidative enzymes [118].

Antioxidants react with free radicals via hydrogen atom transfer (HAT), single electron transfer (SET), or a combination of both of them [119]. The HAT mechanism entails the simultaneous motion of a proton and an electron in a single kinetic reaction step. Free radicals liberate a hydrogen atom from antioxidants, which then become free radicals. The SET mechanism consists of the transfer of electrons from antioxidants to radical cations. The HAT and SET typically occur together. The mechanism that takes place is contingent on the structure and solubility of the antioxidant, the partition coefficient, and the polarity of the solvent [120].

**Figure 5:**
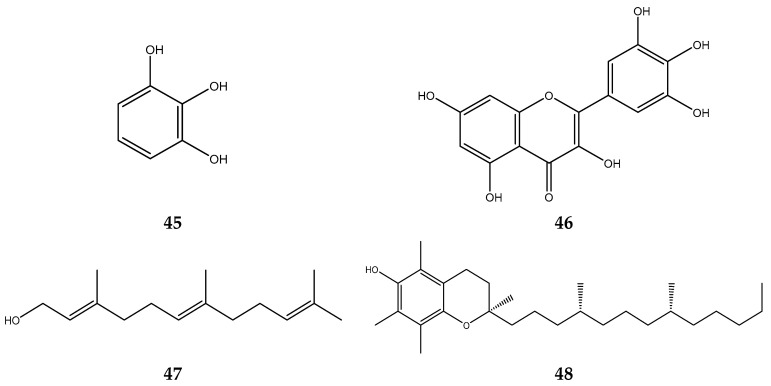
Antioxidant compounds of S. polyanthum essential oil: (**45**) pyrogallol; (**46**) myrecitin; (**47**) farnesol; and (**48**) tocopherol.

*Sygzinium polyanthum* with its synonym *Eugenia Polyantha* contain active compounds as antioxidants [121]. Compounds in *S. polyanthum* acting as antioxidants are pyrogallol, gallic acid, myrecitin, farnesol, phytol, and tocopherol [122,123]. The antioxidant compounds in *S. polyanthum* are shown in Figure 5. 

**Figure 6:**
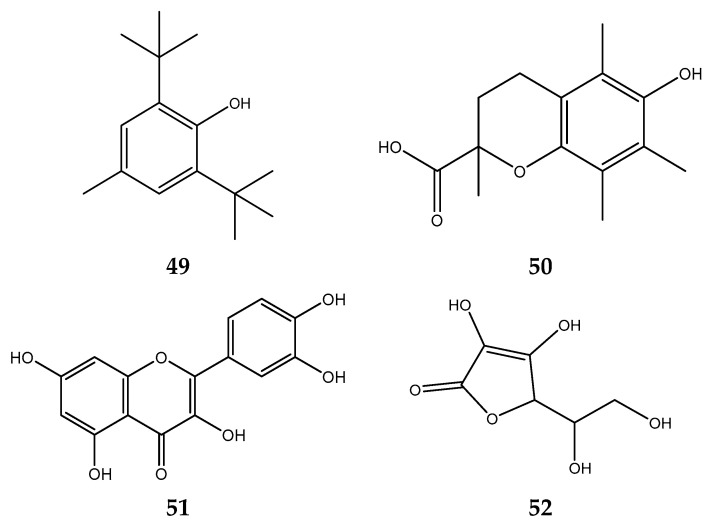
Antioxidant reference compounds of antioxidant analysis: (**49**) butylated hydroxytoluene; (**50**) trolox; (**51**) quercetine; and (**52**) ascorbic acid.

Determined by the number and location of hydroxyl groups and benzene rings present in their molecules, phenolic compounds’ radical scavenging capacity is proportional to their antioxidant activity. Consequently, polymeric structures with a high number of hydroxyl groups and benzene rings possess a greater antioxidant potential [124,125], as shown in Figure 6, which is the typical configuration for evaluating antioxidant activity. Multiple studies have revealed a correlation between total phenolic content and antioxidant activity. Increasing overall phenolic content promotes antioxidant activity [126]. Table 1 shows *S. polyanthum* as an antioxidant in various evaluation methods. 

### 5.2. Antibacterial Activity of S. polyanthum Leaves 

**Figure 7:**
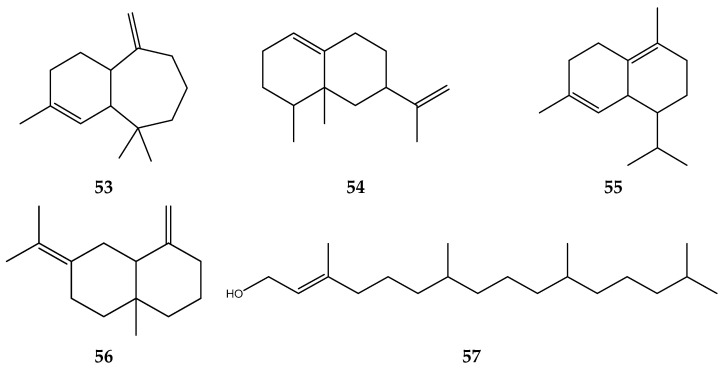
Terpenoid antibacterial compounds: (**53**) α-himachalene; (**54**) eremophilene; (**55**) δ-cadinene; (**56**) γ-selinene; and (**57**) 3,7,11,15-tetramethyl-2-hexadecen-1-ol.

Essential oils, organic acids, bacteriocins, natural polymers, and biosurfactants are antimicrobial agents that can be employed, and some of them have been studied recently. Utilizing natural antimicrobial chemicals without compromising food quality (such as color, texture, and sensory acceptability) has become a new trend in food production and a viable method of quality control [133]. *S. polyanthum* leaf extract inhibits the growth and colonization of various harmful bacteria [134]. Terpenoids were the chemical compounds in the leaves that acted as an antimicrobial. The terpenoids contained within are α-himachalene, eremophilene, δ-cadinene, γ-selinene, and 3,7,11,15-tetramethyl-2-hexadecen-1-ol [135]. The structure of the terpenoids in *S. polyanthum* is shown in Figure 7. 

Since protein is important for bacterial living systems and physiological functions, its loss could suggest an abnormal cell membrane shape and result in malfunction or cell death. The enhanced extracellular protein content of *B. cereus* and *E. coli* treated with curcumin demonstrates the breakdown of cellular membrane integrity. The intracellular ATP concentration and relative fluorescence decreased in response to varied curcumin concentrations, which may have been due to intracellular ATP leakage and depletion of the intracellular ATP pool. In general, intracellular ATP content was regarded as a major element capable of supplying energy for regular physiological processes of the cell [136,137]. These findings revealed a link between the depletion of ATP and the deterioration of cell membrane integrity [138].

Its phytochemical abundance, including flavonoids, tannins, alkaloids, phenols, saponins, steroids, and triterpenoids, contributed to *S. polyanthum* antibacterial activity. As secondary metabolites, flavonoids can suppress bacterial growth. By causing damage to cell membranes and preventing the creation of bacterial cell macromolecules, flavonoids impede the growth of bacteria [139]. Their antibacterial role can be affected by the group of chemicals contained within. The polyphenol group damages the cytoplasmic membranes of bacteria, triterpenoids break down cell membranes, flavonoids obstruct bacterial cell membrane integrity, and alkaloids impact interbacterial osmotic pressure [140]. Diffusion of the cell membrane causes the bacterial membrane destruction in a manner directly equivalent to the added antibacterial concentration [141]. The membrane becomes permeable to cytoplasmic components, resulting in cell death. It was also hypothesized that large quantities of *S. polyanthum* leaf extract contribute to the rapid destruction of microorganisms by causing membrane deterioration and cell wall degeneration. To kill microorganisms, leaf extract must bind, occupy, and remain at the target region for a significant amount of time to impede the metabolic process and chemical reactions of the bacteria. Increasing plant extract can also saturate the target location and produce a quick bactericidal effect [142,143]. The hydrophobicity of plant extracts and their bioactive components contribute to the breakdown of membrane cell lipids, making them more permeable to penetration. In addition, the bioactive components in the extract may impede the enzymatic process necessary for the creation of important metabolites. Bioactive chemicals that interfere with and modify the structure of the ribosome can also inhibit protein synthesis in microorganisms. This interference could cause a misinterpretation of the genetic information encoded on the messenger RNA of bacteria [144].

Mainly, bacteria cell walls are structured by peptidoglycan layers. Antibacterial substances will inhibit the enzyme in the peptidoglycan bacterial cell wall [136]. Gram-positive bacteria are more inhibited by plant essential oil than Gram-negative ones. The plasma membrane is the most frequently reported as antimicrobial target; nevertheless, current research indicates intracellular sites for at least some peptides. Gram-negative bacteria have a lipophilic outer membrane that is phospholipids rich, making it difficult for certain phenolic compounds to permeate the cell wall. Additionally, the presence of periplasmic enzymes in bacteria damages molecules from the outside that enter the bacterial cell. Despite the fact that the majority of antimicrobials peptide work by nonspecific methods, they frequently exhibit selectivity between various microorganisms, such as Gram-negative versus Gram-positive bacteria and susceptibility of fungal cells versus other eukaryotic cells [145,146]. Antimicrobial activity can be determined by measuring the minimum inhibition concentration (MIC) and the minimum bactericidal concentration (MBC) or minimum fungal concentration (MFC). MIC is the minimal antimicrobial agent concentration that inhibits microbial growth, whereas MBC or MFC is the minimal antimicrobial agent concentration necessary to kill microorganisms [147].

*S. polyanthum* leaves have antibacterial activity to *B. cereus* [148], *S. aureus, S. mutans* [149], *Salmonella typhimurium, Escherichia coli*, and *Lactobacillus acidophilus* [134]. In addition to its potential as an antibacterial agent, *S. polyanthum* also possesses antifungal properties. Antifungal properties of plants are typically derived from their secondary metabolites. Common active antifungal components in plants include phenolic compounds, hexanal, hexanol, glucosinolate, and essential oils [50]. Antifungal mechanisms occur in fungal cells as a result of ergosterol inhibition induced by 5,6 desaturase (ERG3) inhibition [150,151]. Ergosterol is the most prevalent sterol in the fungal plasma membrane, and its binding to an ergosterol-specific enzyme can result in lanosterol demethylation [152]. The antimicrobial capacity of *S. polyanthum* is shown in Table 2. 

## 6. Natural Food Preservation

Globally, food quality and safety has become a major public concern, and dependable sensing or monitoring systems are required to confirm the values of various food stuffs, especially perishable foods such as pork, seafood, lamb, beef, and poultry [150]. Numerous foods are naturally perishable and must be protected from spoilage during preparation, storage, and distribution in order to achieve their intended shelf life. Because food products are now frequently sold in distant regions worldwide, the need for these products to have long shelf-lives has also increased. The need to extend the shelf-life of food triggered the development of food preservation techniques [162]. Some of the most prevalent preservation techniques include food additive utilization [163], chilling technology [164], and advance packaging technology [24,165,166].

In addition to healthful food, people are increasingly demanding safer alternatives to the use of synthetic additives in the food industry. As a result, numerous plant-based preservatives have been evaluated by the food industry [167]. Some plants contain phytochemicals with antimicrobial and antioxidant properties; consequently, they can preserve and prolong the shelf life of food. In addition to preserving food by inhibiting bacterial activity, herb extracts rich in phenolic compounds can also preserve food by inhibiting lipid oxidation due to their high potential antioxidant activity [168]. As a result, plant-based preservatives have been researched and incorporated into food as a replacement for synthetic preservatives [169]. As seasonings, flavor enhancers, perfumes, and food preservatives, spices are a class of plants. Some companies add synthetic antimicrobial compounds such as benzoate, nitrate, and nitrite to food to prevent food spoilage caused by the growth of microbes [170].

By removing all pro-oxidants and air, together with inactivating all enzymes involved in food degradation, it is possible to prevent or reduce food spoilage [171]. Several food groups that have the potential to be preserved based on the synergistic activity of herbs as antioxidants and antibacterials include fish and meat products, bakery products, dairy products, vegetable and fruit juices, sauces, cereals, vegetables and fruits, and oils [172].

### 6.1. Food Antioxidant Preservative

Antioxidants are added to food to prevent the oxidation that causes rancidity and browning. Antioxidants are commonly utilized as preservatives in the food industry. By including antioxidants into diets rich in unsaturated fats, rancidity can be avoided [173]. In foods, antioxidants are used to reduce lipid oxidation and development by quenching free radicals. These highly effective antioxidants contain one or more hydroxyl groups or phenol [174]. In the food matrix, PUFAs are often present as a lipid phase dispersed in an aqueous solution covered with a surfactant or emulsifier, which generates a thin interfacial layer that separates oil and water, or what is widely known as an oil-in-water emulsion. This renders the food emulsion unstable because it is easily exposed to air, oxygen, or light, which causes the food to oxidize quickly [175]. Applying antioxidants such as phenols to prevent oxidation is a highly efficient and cost-effective strategy. By rebuilding the parent fatty acids and producing less reactive radicals, ArO•, phenol compounds effectively neutralize peroxide radicals, LOO• [176,177]. Antioxidants are necessary for avoiding the oxidative damage of EPA and DHA [178]. When quercetin, ascorbic acid, and curcumin were added to the oil-in-water emulsion system, the peroxide value of the oil decreased significantly. This demonstrates the presence of the “polar paradox” notion, according to which polar antioxidants tend to dissolve in nonpolar samples. The combination of these antioxidants demonstrates a synergistic effect on the suppression of fat oxidation [179].

To appropriately evaluate the potential of *S. polyanthum* as an antioxidant preservative, models with the chemical, physical, and environmental characteristics predicted in food products must be built, particularly models with a high lipid content. There are three techniques to evaluate antioxidant model systems: bulk oil, oil-in-water emulsions, and muscle foods [180]. Volatile organic acids are byproducts of secondary lipid oxidation, and some of them have extremely low threshold values for offensive odors [181]. Moreover, the lipid oxidation products result in the loss of food nutritional value, texture, color, and certain functional properties [182]. Due to their high fat content, which makes them especially susceptible to lipid oxidation, it is crucial to include antioxidants in these food matrices. However, consumers are unwilling to accept reductions in the product sensory quality (color, odor, or flavor), which is frequently affected by the addition of natural antioxidants [12]. *S. polyanthum* as an antioxidant food additive is described in Table 3. 

### 6.2. Food Antimicrobial Preservative

Natural antimicrobial food preservatives can be obtained from animal (lactoferrin, chitosan, lysozyme, and milk-derived peptide), plant (saponins, flavonoids, carvacrol, thymol, citral, eugenol, linalool, and terpenes), and microbial agents (reuterin, nisin, and pediocin) [193]. The effectiveness of antimicrobial compounds is reliant upon the pH of the food and the type and number of contaminating microorganisms, as well as the type and concentration of antimicrobials compounds. Because the absorption of compounds is related to temperature, storage temperature can also influence antimicrobial efficacy [194]. *S. polyanthum* as an antimicrobial food additive is described in Table 4.

## 7. Conclusions

*S. polyanthum* contains secondary metabolites such as phenolic compounds, flavonoid, tannins, alkaloids, phenols, saponins, steroids, and triterpenoids. Based on its capacity as an antioxidant and the antimicrobial value of *S. polyanthum*, it has moderate antioxidant and antimicrobial potential. The addition of *S. polyanthum* to food is able to extend the food’s shelf-life, though it is not significant. Based on its application in food models, *S. polyanthum* is able to play a role as a moderate natural preservative.

## Figures and Tables

**Table 1:** Antioxidant capacity of *S. polyanthum*.

Solvent	Method	IC_50_	Standard	IC_50_	Reference
Methanol	DPPH	20.90 µg/mL	BHT	18.50 µg/mL	[127]
	FRAP	77.55 µg/mL		Not detected	
	DPPH	2.82 µg/mL			[6]
	DPPH	44.35 µg/mL	Trolox	3.09 µg/mL	[128]
	ABTS	17.69 µg/mL		4.11 µg/mL	
	DPPH	77.06 mg TEAC/g			[129]
	FRAP	7.92 mg TEAC/g			
	ABTS	83.19 mg TEAC/g			
Ethanol	DPPH	10.89 µg/mL	Quercetin	5.24 µg/mL	[130]
	FRAP	27.76 mmol/g		27.03 mmol/g	
Chloroform	DPPH	0.029 mg/mL	Quercetin	0.0080 mg/mL	[131]
	ABTS	1.91 mmol TEAC			
Petroleum ether	DPPH	0.023 mg/mL	Quercetin	0.0080 mg/mL	[131]
	ABTS	0.32 mmol TEAC			
Hexane	DPPH	3121.73 µg/mL	Ascorbic acid	693.30 µg/mL	[132]
Ethyl acetate	DPPH	73.15 µg/mL	Ascorbic acid	3.94 µg/mL	[132]
Essential oil	DPPH	2.08 µg/mL	Ascorbic acid	3.73 µg/mL	[109]
	FRAP	3.28 µg/mL		10.24 µg/mL	

**Table 2:** Antimicrobial capacity of *S. polyanthum*.

Source	Microbial	MIC	MBC/MFC	Reference
Methanol extract	*C. botulinum*	>5000 mg/L	-	[153]
	*Bacillus cereus*	0.31 mg/mL	2.50 mg/mL	[154]
	*Bacillus subtilis*	0.63 mg/mL	2.50 mg/mL	
	*S. aureus*	6.25 mg/mL	-	[155]
	*S. pyogenes*	6.25 mg/mL	-	
	*Methicillin-resistant*	6.25 mg/mL	-	
	*K. pneumiae*	6.25 mg/mL	-	
	*E. coli*	12.15 mg/mL	-	
	*C. albicans*	1.25 µg/mL	1.25 µg/mL	[156]
Ethanol extract	*Shigella dysenteriae*	20% *b*/*v*	20% *b*/*v*	[157]
	*E.coli*	1.25 mg/L	2.50 mg/L	[158]
	*K. pneumoniae*	1.25 mg/L	2.50 mg/L	
	*S. aureus*	0.63 mg/L	1.25 mg/L	
	*S. typhimurium*	1.25 mg/L	1.25 mg/L	
	*S. typhimurium*	0.63 mg/L	0.63 mg/L	
	*C. albicans*	0.16% *w*/*v*	0.16% *w*/*v*	[159]
Water extract	*S. mutans*		30 mg/mL	[160]
Essential oils	*B. subtilis*	31.25 µg/mL	-	[161]
	*E. coli*	>1000 µg/mL	-	
	*S. aureus*	>1000 µg/mL	-	
	*S. typhimurium*	>1000 µg/mL	-	
	*V. cholera*	>1000 µg/mL	-	

**Table 3:** The impact of *S. polyanthum* as an antioxidant food preservative on a variety of foods and observation techniques.

Food	Preservative	Methods	Conclusions	References
Sargassum tea	Simplisia	Water infusion	The addition of *S. polyanthum* contributed to a reduction in IC_50_ concentration. IC_50_ measurements were carried out using the ABTS method.	[183]
Salted egg	Simplisia	Water infusion	The concentration of antioxidant IC_50_ reduced from 89.92 to 88.58 mg/g after soaking salted egg at varying concentrations 0–10%. *S. polyanthum* addition.	[184]
Pork	Simplisia	Water infusion	There is a significant color difference between pork immersed in 0 and 10% *S. polyanthum* leaf. After soaking *S. polyanthum* for 6 h, significant differences were also observed. Another significant difference between the control and 5% *S. polyanthum* immersion was the difference in meat texture.	[185]
Beef	Simplisia	Water infusion	Various concentrations until 15% of *S. polyanthum* leaf infusion had significant effects on the odor, color, texture, shelf life at room temperature, pH, and water content of Bali beef but had no significant effect on the beef’s ability to retain water.	[186]
Beef sausage	Extract	Extract addition	*S. polyanthum* possesses antioxidative properties and could be utilized as a natural antioxidant to prevent lipid oxidation and oily food products. The addition of *S. polyanthum* at a concentration of 1.50 ppm inhibits oxidative damage in beef sausages.	[187]
Bulk cooking oil	Extract	Extract addition	The addition of the ethyl acetate fraction lowered the peroxide values. The addition of 1.0% *S. polyanthum* lowered the peroxide value of bulk cooking oil from 7.75 to 5.04 meq O_2_/kg. Using 0.2% TBHQ as a control, the peroxide value of bulk cooking oil was reduced to 4.14 meq O_2_/kg.	[188]
Bulk cooking oil	Extract	Extract addition	The optimal amount of *S. polyanthum* extract added to cooking oil is 0.8%. The addition of *S. polyanthum* altered the iodine value and acid value from 42.9 to 48.2 g I_2_/100 mL and from 0.42 to 0.34 KOH/g, respectively. As 0.2% TBHQ was added as a control, the iodine value and acid value changed to 48.7 g I_2_/100 mL and 0.19 KOH/g.	[189]
Meat	Extract	Extract addition	The tested extracts were methanol, methanol–water, and water. Using the TBARS technique, the addition of 3% *S. polyanthum* extract to raw and cooked meat inhibited fat oxidation. The meat samples were stored at 4 °C for seven days prior to testing. Based on the results of the tests, it was determined that the best extract to inhibit fat oxidation in meat was water extract. The lipid protections of water extract on raw meat and cooked meat are up to 58 and 68%, respectively.	[190]
Salted egg	Extract	Extract addition	The concentration of extract addition on salted egg duck media is 25 and 50%. The antioxidant capacity of standard salted eggs, 25% extract addition and 50% extract, were measured to be 4.45, 30.85, and 44.32%. The quality of salted eggs is similar to that of standard salted eggs, despite an increase in albumin index and Haugh unit values to 0.053–0.060 and 44–47, respectively.	[191]
Jelly candy	Extract	Extract addition	*S. polyanthum* extract addition decreases jelly oxidation by up to 50%. Experiments are held for up to 12 days of storage.	[192]

**Table 4:** The impact of *S. polyanthum* as an antimicrobial food preservative on a variety of foods and observation techniques.

Food	Preservative	Methods	Conclusions	References
Chicken meat	Simplicia	Water infusion	Soaking and storage time had no effect on the physical characteristics of chicken meat, but it could reduce the color value of raw meat while increasing the aroma value, as well as the tenderness and aroma of cooked meat. Soaking chicken meat in *S. polyanthum* leaf infusion with varying storage times can increase tenderness and inhibit microbial growth until the fourth day. However, *S. polyanthum* leaf infusion had no effect on the pH or cooking loss of chicken meat. *S. polyanthum* infusion can reduce the total number of microbes in chicken meat during refrigerator storage.	[195]
Chicken meat	Extract	Water infusion	Variations in the concentration of *S. polyanthum* leaf infusion and the length of observation at room temperature had a substantial impact on the total number of bacteria in fresh chicken meat.	[196]
Chicken meat	Simplicia	Water infusion	At the optimal concentration of 10%, *S. polyanthum* leaf infusion can inhibit bacteria growth on chicken meat during storage, extending its shelf life by up to three days at 3–7 °C.	[197]
Shrimp and chicken	Extract	Dilution	Chicken and shrimp were treated with *S. polyanthum* leaf extract at various concentrations, 0.0, 0.1, and 1.00%, and exposure periods of 5 and 10 min. In untreated chicken samples, *S. aureus* TPC values were determined to be 6.66 and 8.66 CFU/mL. In untreated shrimp samples, *S. aureus* TPC values were determined to be and 7.25 and 6.54 CFU/mL. However, neither sample contained *E. coli, Salmonella spp.,* or *Vibrio cholerae*. The number of *S. aureus* TPCs in chicken meat and shrimp began to decrease significantly after 5 min of exposure to *S. polyanthum* leaf extract at a concentration of 0.01%. There were no statistically significant differences between exposure times. TPC was reduced from 6.66 to 0.00 CFU/mL and from 8.66 to 4.88 CFU/mL in shrimp, whereas TPC *S. aureus* was reduced from 7.25 to 3.88 CFU/mL and from 6.54 to 4.92 CFU/mL in chicken and shrimp, respectively, following treatment with 1.0% extract for 10 min.	[198]
Tilapian fish	Extract	Extract addition	Using 15% *S. polyanthum* extract and storing for seven days was able to maintain the number of bacterial colonies below the national regulator requirement.	[199]
Dug eggs	Simplicia	Water infusion	Using 5% (*v*/*v*) S. polyanthum addition to salted duck eggs can inhibit *Proteus mirabilis* growth significantly. It is shown by the total plate number decreasing.	[200]
Tofu	Essential oil	Water infusion	Essential oil is added to tofu bacterial growth media. Essential oil concentrations are 0.063, 0.313, and 1.563 mg/mL. Tofu and bacteria are incubated for 2, 4, 6, and 8 days. Bacterial growth is monitored visually. Preservation of essential oil is equal to its concentration. Essential oil optimally preserved tofu in 6-day incubation.	[161]

## Data Availability

The study did not report any data.
